# PARIHS revisited: from heuristic to integrated framework for the successful implementation of knowledge into practice

**DOI:** 10.1186/s13012-016-0398-2

**Published:** 2016-03-10

**Authors:** Gill Harvey, Alison Kitson

**Affiliations:** 1 School of Nursing, University of Adelaide, Adelaide, SA 5005 Australia; 2 Alliance Manchester Business School, University of Manchester, Manchester, UK; 3 Associate Fellow, Green Templeton College, University of Oxford, Oxford, UK

**Keywords:** PARIHS, i-PARIHS, Implementation framework, Facilitator role, Facilitation

## Abstract

**Background:**

The Promoting Action on Research Implementation in Health Services, or PARIHS framework, was first published in 1998. Since this time, work has been ongoing to further develop, refine and test it. Widely used as an organising or conceptual framework to help both explain and predict why the implementation of evidence into practice is or is not successful, PARIHS was one of the first frameworks to make explicit the multi-dimensional and complex nature of implementation as well as highlighting the central importance of context. Several critiques of the framework have also pointed out its limitations and suggested areas for improvement.

**Discussion:**

Building on the published critiques and a number of empirical studies, this paper introduces a revised version of the framework, called the integrated or i-PARIHS framework. The theoretical antecedents of the framework are described as well as outlining the revised and new elements, notably, the revision of how evidence is described; how the individual and teams are incorporated; and how context is further delineated. We describe how the framework can be operationalised and draw on case study data to demonstrate the preliminary testing of the face and content validity of the revised framework.

**Summary:**

This paper is presented for deliberation and discussion within the implementation science community. Responding to a series of critiques and helpful feedback on the utility of the original PARIHS framework, we seek feedback on the proposed improvements to the framework. We believe that the i-PARIHS framework creates a more integrated approach to understand the theoretical complexity from which implementation science draws its propositions and working hypotheses; that the new framework is more coherent and comprehensive and at the same time maintains it intuitive appeal; and that the models of facilitation described enable its more effective operationalisation.

**Electronic supplementary material:**

The online version of this article (doi:10.1186/s13012-016-0398-2) contains supplementary material, which is available to authorized users.

## Background

In 2008, the PARIHS group published a paper in Implementation Science that summarised the work over the previous 10 years in developing and refining the PARIHS (Promoting Action on Research Implementation in Health Services) framework [1]. From its inception, PARIHS argued that successful implementation (SI) of evidence into practice was a function of the quality and type of evidence (E), the characteristics of the setting or context (C) and the way in which the evidence was introduced or facilitated (F) into practice. Each of these dimensions was further subdivided into a number of sub-elements that needed to be considered in order for implementation to be successful [2, 3].

The 2008 paper outlined three linked areas of work in developing PARIHS, namely, conceptual development [4–6], empirical testing and refinement [7] and the development of reliable measures to diagnose and evaluate an organisation’s readiness for change and the effectiveness of that change [8–10]. It concluded by identifying a number of challenges including the need for more theoretical work on the conceptual framework, the need to set up more rigorous ways to develop and test the diagnostic and evaluative methodologies and associated instruments based on elements of PARIHS and the need to agree upon the practical contents of a facilitator training programme that would equip facilitators to know how to operationalise the framework. Subsequently, both PARIHS team members [11–13] and other research teams [14, 15] have been involved in studies to evaluate and refine the framework further. These studies have reinforced some of the conclusions of the 2008 paper and identified some additional issues for consideration. For example, Helfrich and colleagues [14] undertook a critical synthesis of the literature on PARIHS and identified a number of perceived limitations to its effective utilisation. These included the lack of evidence from prospective implementation studies on its effectiveness; lack of clarity between elements and sub-elements of the framework; a predominant focus on the facilitation role rather than the facilitation process and the lack of a clear definition of what successful implementation actually was. Building on this review, a revised PARIHS framework was put forward, including a detailed diagnostic tool based on the refined elements and sub-elements of the framework [15].

A repeat search in 2014 using the same databases and search terms as the review by Helfrich and colleagues in 2010 [14] identified over 40 more papers that reported applying PARIHS [16]. This indicates continuing interest in using the framework and reinforces what Helfrich and colleagues observed in terms of the framework’s intuitive appeal and relevance to the real world setting. However, prospective studies remain limited. One exception to this is a prospective study on peri-operative fasting, which used PARIHS to design a pragmatic trial to test the effectiveness of the introduction of guidelines to improve practice [12, 17]. From their analysis, the authors suggested that an additional weakness in the framework was the failure to acknowledge the central role of individuals in determining the process and outcomes of implementation, mediated through individual interactions with and influence on the evidence and context dimensions of PARIHS. Useful findings have also emerged from reviews that have compared PARIHS to other implementation frameworks and models. Tabak and colleagues reviewed over 60 models and frameworks and suggested that PARIHS lacked a focus on the system and policy level of implementation [18]. Flottorp and colleagues also undertook a review of frameworks and their findings indicated that PARIHS failed to pay attention to the individual health professional and the wider social, political and legal context of implementation [19].

Our own ongoing application of the framework in implementation studies (see, for example, [11, 13, 20–22]) together with critiques and evaluations of the framework by other research teams has led us to create a refined version of PARIHS. It is called the integrated or i-PARIHS framework. This paper describes the revised framework, outlines the new elements and explains why the changes have been made. Within this discussion, we draw on empirical data from three case studies of implementation (summarised in Table 1). The paper then describes how the i-PARIHS framework can be operationalised and summarises the underpinning theoretical antecedents of the framework. We conclude the paper by raising some questions for further consideration and outlining plans for future research and development activity.

**Table 1:** Three implementation case studies

Implementation study	Innovation	Recipients	Context	Facilitation	Implementation outcomes
1. Improving the identification and management of chronic kidney disease (CKD) in primary care	Starting point: existing data indicating prevalence levels of CKD in the local population were lower than would be expected National clinical guideline presenting evidence-based recommendations for identifying and managing CKD Stakeholder group convened to consider the evidence and the local population data; identified 2 targets for improvement	General practice teams recruited to participate in an improvement collaborative; each team required to have multi-disciplinary membership Sponsorship from senior leaders in the primary health care setting Some resistance encountered at a local level, e.g. from practice colleagues who did not recognise CKD as a priority, were uncomfortable disclosing to patients or did not feel sufficiently involved	Practices were working to a pay-for-performance system; CKD was part of this system; hence, there was an incentive to improve Wider changes occurring in relation to the organisation and management of general practice	Facilitation teams set up, comprising a mix of internal and external novice, experienced/expert facilitators, supported by clinical leaders and project managers Facilitation methods used included collaborative learning events, local context assessment, Plan-Do-Study-Act (PDSA) cycles, audit and feedback, benchmarking of data and regular practice visits	Before and after study design Recorded prevalence of CKD increased by 1.2 % in 30 participating practices (*n* = 1863 additional patients with CKD identified) compared to a national increase of 0.2 % Management of blood pressure improved in line with national guidelines from 34 to 74 % (cohort 1) and 58 to 83 % (cohort 2) [21]
2. Improving continence care in a nursing home setting	Starting point: 4 evidence-based recommendations for practice identified from an international clinical guideline by the project stakeholder group Recommendations were discussed and reviewed by facilitators and a set of common audit criteria agreed	Facilitators were encouraged to establish improvement teams within the nursing home Some difficulties in convincing colleagues that improvements in continence of long-term residents was possible Input from continence nurse specialist Use of patient stories to highlight the need/potential for improvement Gate-keeper role of nursing home manager	Contextual challenges in a number of homes caused by change of management and reorganisation Culture of managing incontinence rather than promoting continence Positive impact of external inspection/accreditation	Internal novice facilitators trained and supported by external expert facilitators Internal facilitators encouraged to partner with a buddy—some did and others did not Majority of external support provided virtually Facilitation methods: joint training, monthly teleconference meetings, audit and feedback and PDSA cycles	Cluster RCT showed no difference between control and intervention wards on primary outcome measure of overall compliance to continence recommendations [11, 85] but significant improvements on a number of secondary outcomes and 1 of the 4 specific recommendations Internal evaluation demonstrated variable achievement of key audit targets by participating sites [45]
3. Improving nutritional care of older adults in an acute care setting	Starting point: evidence review to identify three interventions to be implemented as part of the project Combined the three interventions (nutritional screening, nutritional supplements and red tray system) into an improvement bundle	Organisation wide approach adopted, with senior leadership support and communication strategy in place Dietitians previously tried to introduce improvements but unable to secure buy-in Formed part of an inter-disciplinary team in this project with involvement of other clinical colleagues and other departments such as catering and supplies	Contextual issues to be negotiated at an organisational level related to the infrastructure and resources required to enable implementation, e.g. providing fridges at ward level, financing the purchase of nutritional supplements, issues of supply and stock management	Experienced internal facilitators supported by external expert facilitators Internal facilitators recruited ward level clinical champions to work with them Facilitation methods: staff information and education programmes, audit and feedback	Stepped wedge RCT [86] demonstrated no difference in weight loss after 1 week between intervention and control wards Improvement noted on key audit measures relating to nutritional screening, provision of nutritional supplements and use of red trays for patients requiring assistance with feeding [46]

## Main Text

The main reasons for re-visiting the original PARIHS framework included:

- The original framework failed to address key dimensions, including the intended targets for implementation and the wider external context (social, political, policy and economic) in which implementation occurs [14, 18, 19]
- Growing evidence on the key role individuals play in the implementation process [12]
- Increased interest and awareness of relevant theories that can and should inform implementation strategies [23–25]
- Recognition of the diverse ways in which people were applying PARIHS, not simply to guide the implementation of more conventional research evidence in the form of clinical guidelines or evidence summaries, but to inform and evaluate developments in practice more generally [26]

Based on our analysis of these issues, we are proposing the revision of the key constructs of evidence, context and facilitation and suggesting the addition of a new construct termed ‘recipient’. The original PARIHS framework was expressed as a simple equation (Table 2). Critics have rightly pointed out that we did not define what successful implementation meant [14, 15]. In our revised, i-PARIHS framework, successful implementation is primarily specified in terms of the achievement of implementation/project goals and results from the facilitation of an innovation with the recipients in their (local, organisational and health system) context (Table 2). The core constructs of the i-PARIHS framework are facilitation, innovation, recipients and context, with facilitation represented as the active element assessing, aligning and integrating the other three constructs. As illustrated, a number of other characteristics of successful implementation are proposed, reflecting the multi-dimensional nature of the constructs.

**Table 2:** From PARIHS to i-PARIHS (adapted from [16])

‘Successful implementation’ in the original PARIHS framework	‘Successful implementation’ in the revised i-PARIHS framework
SI = ƒ(E,C,F) SI = successful implementation ƒ = function (of) E = evidence C = context F = facilitation	SI = Fac^n^(I + R + C) SI = successful implementation Achievement of agreed implementation/project goals The uptake and embedding of the innovation in practice Individuals, teams and stakeholders are engaged, motivated and ‘own’ the innovation Variation related to context is minimised across implementation settings Fac^n^ = facilitation I = innovation R = recipients (individual and collective) C = context (inner and outer)

### The innovation construct

The original PARIHS construct of evidence adopted a broad view of evidence, comprising information from research, alongside clinical, patient and local experience [6]. In i-PARIHS, we have further extended the construct to embrace a more explicit view of how the characteristics of knowledge affect its migration and uptake in different settings. This includes the more emergent, inductive ways in which evidence is generated from practice as, for example, within practice development initiatives in nursing and healthcare [27–29]. Our proposition is that people rarely take evidence in the original form of a systematic review or clinical guideline and directly apply it within an implementation project rather they incorporate evidence in a number of different ways, which typically involves adapting the original evidence in some way to suit their particular situation, a process described by some as ‘tinkering’ [30] whereby explicit knowledge is blended with tacit, practice-based knowledge.

This is clearly apparent in one of the cases we draw on in this paper, namely a project to improve the identification and management of chronic kidney disease (CKD) in a healthcare region in England [21, 31]. Aware of the potential to improve CKD, the team leading the project accessed a recently produced national clinical guideline on the identification and management of CKD in primary care [32]. However, rather than setting out to ‘implement the guideline’, a number of prior processes were put in place. Firstly, a local stakeholder group comprising patient representatives, clinicians from acute and primary care, researchers and managers was established to consider the evidence and agree on the priorities at a local level. This involved taking into consideration existing policies and practice at the local level, including the CKD related measures in the national pay-for-performance system in primary care and the local rates of achievement on these indicators. From the stakeholder deliberations, a decision was made to distil the evidence from the guideline into two overarching aims related to improving the identification of CKD patients within a practice population and, once identified and on a practice register, to improve the management of patient blood pressure to evidence-based targets.

This process of aligning external explicit evidence with local priorities and practice is an important way of enhancing the compatibility of a proposed change, as recognised in the innovation literature [33–35]. For these reasons, we have re-labelled the construct ‘innovation’, incorporating Rogers’ seminal work on the diffusion of innovations [33, 34] and other key studies on the nature of innovation within and outside healthcare [35, 36]. We argue that evidence is one type of knowledge and (new) knowledge is the substance that needs to be introduced in order to generate change and improvement. The characteristic of the knowledge creates a set of conditions that make it more or less likely to be recognised and applied. This phenomenon is well described in Roger’s Diffusion of Innovations Theory [33, 34], for example, in terms of the likely fit of the new knowledge with existing practice, the relative advantage it presents and potential trialability. We are therefore proposing ‘innovation’ as a central construct within the i-PARIHS framework but with an explicit focus on sourcing and applying available research evidence to inform the innovation. Table 3 summarises the main characteristics of the innovation to be considered in implementation.

**Table 3:** Characteristics of the innovation, recipients and context to be considered within the i-PARIHS framework

Innovation	Recipients	Context
Underlying knowledge sources Clarity Degree of fit with existing practice and values (compatibility or contestability) Usability Relative advantage Trialability Observable results	Motivation Values and beliefs Goals Skills and knowledge Time, resources, support Local opinion leaders Collaboration and teamwork Existing networks Power and authority Presence of boundaries	Local level: Formal and informal leadership support Culture Past experience of innovation and change Mechanisms for embedding change Evaluation and feedback processes Learning environment Organisational level: Organisational priorities Senior leadership and management support Culture Structure and systems History of innovation and change Absorptive capacity Learning networks External health system level: Policy drivers and priorities Incentives and mandates Regulatory frameworks Environmental (in)stability Inter-organisational networks and relationships

### The recipient construct

This is a new construct, added in response to consistent feedback that insufficient attention had been paid in the original framework to the actors involved in implementation. Although reviews and empirical studies applying PARIHS have emphasised the importance of the individual on implementation processes and outcomes [12], we are proposing recipients as a construct that encompasses the people who are affected by and influence implementation at both the individual and collective team level. This extension enables the i-PARIHS framework to consider the impact individuals and teams have in supporting or resisting an innovation. We have elected to consider recipients at both an individual and collective level as alongside research highlighting the importance of individuals in supporting or resisting change [12, 37]; there is good evidence to suggest that groups or teams of individuals have an important role in determining the uptake of new knowledge in practice. This is particularly evident in studies that have been undertaken on communities of practice and the notion of collective ‘mindlines’ influencing the uptake (or not) of evidence in practice [38–40].

The CKD case study illustrates one way in which actors at the local level can influence the course of implementation, as one of the challenges encountered was whether practice staff perceived value in ‘labelling’ patients with CKD. This was particularly the case for older patients as some General Practitioners (GPs) and practice nurses viewed declining renal function as a natural part of ageing and believed that disclosing a diagnosis of CKD could cause unnecessary anxiety in patients. By adopting this approach, opportunities to improve self-management and overall management of cardiovascular disease and to address the issue of increased susceptibility to acute kidney injury were potentially missed [41].

A second case study which elucidated the key role of recipients focused on the implementation of evidence-based recommendations for the management of continence in a nursing home setting [42]. Focusing on goals to improve the assessment and attainment of continence amongst residents, a key area was addressing care staffs’ strongly held views as to whether such goals were achievable, particularly where residents had been managed as ‘incontinent’ over prolonged periods of time. This required a significant amount of effort to change the mindset amongst nursing home staff about achieving continence. Various strategies were helpful in this regard, including input, support and practical guidance from a continence nurse specialist. In one nursing home, the staff responsible for facilitating implementation collected stories from residents about their experience of living with incontinence, which provided a very powerful motivational tool to convince their colleagues of the need to change.

As these examples illustrate, the people involved in implementation, including their views, beliefs and established ways of practice, can significantly affect the ease of introducing an innovation or change. A wide range of stakeholders potentially fit into the construct we have labelled ‘recipients’ including patients and clients, clinical staff and managers. It is also apparent that the relationship between the innovation and the recipients is in many ways an inter-dependent one. Given this set of circumstances, part of the facilitator’s role at the recipient level involves assessing the actual and potential boundaries that exist and the ways in which these barriers might exert an influence during implementation [43]. Table 3 identifies the main characteristics of the recipients at the individual and collective level.

### The context construct

Context remains a core construct within i-PARIHS but with a wider focus on the different layers of context, from the micro through the meso and macro levels, that can act to enable or constrain implementation. In the PARIHS framework, we defined context in terms of resources, culture, leadership and orientation to evaluation and learning; however, we did not delineate between the immediate local context and the wider organisational context. Furthermore, we did not explicitly consider the impact that the wider health system—the external context—could have on implementation processes and outcomes. These meso and macro level contextual factors have been recognised as important considerations, for example, in other implementation frameworks such as the Consolidated Framework for Implementation Research [44] and in reviews of models and theories of implementation [19]. We have also observed their influence in our own empirical research. For example, in the case study on promoting continence, in some of the countries studied, a focus on continence formed part of the external accreditation system for nursing homes. This created a driver for introducing changes to improve the management of continence at a local level [45]. Similarly in the case study of CKD, the presence of CKD indicators in the pay-for-performance system in primary care created an incentive for improvement [21].

In a third case study that focused on the prevention and reduction of weight loss amongst older patients in an acute hospital setting, a number of contextual factors were important, particularly at the organisational level [46]. The implementation project introduced three evidence-informed interventions, one of which was the provision of oral nutritional supplements for older patients at risk of malnutrition. However, the reality of making these supplements available at the point of care delivery required the agreement of financial support to make the supplements, and the fridges to store them in, available in the ward setting. Furthermore, negotiations with the stocks department were needed to address the issue of stock supply and management. These are typical of the sort of organisational context issues that have to be considered within the process of implementation.

Consequently, in the i-PARIHS framework, we have made a distinction between the layers of inner and outer context, where inner context includes both the immediate local setting, whether a ward, unit, hospital department or primary care team, and the organisation within which this unit or team is embedded. Outer context refers to the wider health system in which the organisation is based and reflects the policy, social, regulatory and political infrastructures surrounding the local context. Table 3 illustrates the differentiation of inner and outer context at the micro, meso and macro levels.

### The facilitation construct

As with the original PARIHS framework, facilitation remains a core construct. However, we emphasise facilitation as the active ingredient within i-PARIHS by positioning it differently to the other main constructs of innovation, recipients and (inner and outer) context (Table 2). We propose that facilitation is the construct that activates implementation through assessing and responding to characteristics of the innovation and the recipients (both as individuals and in teams) within their contextual setting. This requires a role (the facilitator) and a set of strategies and actions (the facilitation process) to enable implementation. The i-PARIHS framework therefore locates the success or otherwise of implementation upon the ability of the facilitator and the facilitation process to enable recipients within their particular context to adopt and apply the innovation by tailoring their intervention appropriately.

We have adopted this position for a number of reasons, both experientially and empirically based. Tracing the history of facilitation as a concept in healthcare [5], there is a tradition of applying facilitator roles to support the implementation of changes in practice. From the introduction of facilitators to promote primary care prevention programmes in the 1980s [47], the use of facilitators in primary care has become commonplace, particularly supporting the implementation of change through quality improvement methods [48–51]. A 2012 systematic review of practice facilitation in primary care concluded that practices supported by a facilitator were 2.76 times more likely to adopt evidence-based clinical guidelines [52]. Within the 23 studies reviewed, facilitators employed a number of different facilitation strategies, in particular audit and feedback (used in 100 per cent of studies) and interactive consensus building and goal setting (91 per cent use), alongside reminders, tailoring to context and quality improvement tools such as Plan-Do-Study-Act (PDSA) cycles. This concurs with our own experiences of applying facilitation to support the development of standards, audit and quality improvement in nursing and health care [53, 54]. More recently, the use of facilitation has been evaluated in a number of other settings. For example, the NeoKIP (Neonatal Knowledge into Practice) trial evaluated the effectiveness of facilitation as a knowledge translation intervention for improved neonatal health and survival [55]. Using lay members of the community who received training in facilitation techniques such as PDSA and group consensus building, the study demonstrated a reduced neonatal mortality of 49 % in the third year of the intervention [56]. In the United States Veterans Health Administration, a number of studies have demonstrated the benefits of using facilitation to support the implementation of evidence into clinical practice (for example [57, 58]), whilst in the UK, facilitators have been employed to support the implementation of evidence-based vascular care [20, 21], as described in the CKD case study.

To fulfil the role effectively, facilitators have to be able to function in a flexible and responsive way to tailor their approach to the particular issue, setting and people involved; hence, our proposition that facilitation comprises the active element of implementation. However, as the case studies in Table 1 illustrate, evidence from effectiveness studies of facilitation is mixed. This likely reflects the fact that facilitation itself is a complex intervention, involving one or more individuals in the role of facilitator, applying a combination of improvement and team-focused strategies to enable and support change. In some cases, facilitators are internal to the implementation setting; in others, they are external and sometimes a combination of internal and external facilitators is used. Studies that report process evaluation alongside effectiveness data demonstrate the importance of having the right individuals in the role with the right level of skills, knowledge, support and mentoring [59, 60]. This highlights the need to consider issues of facilitator recruitment, selection, preparation and development when designing and conducting implementation studies that employ facilitation as an intervention. These are issues that we have taken into consideration in our proposed operationalisation of facilitation within i-PARIHS.

### How the i-PARIHS framework is actioned

A consistent criticism of the original PARIHS framework was that it was difficult to operationalise [15]. In developing the i-PARIHS framework, we have used ongoing empirical research from our own and other teams’ application, development and evaluation of PARIHS to present a practical model of facilitation (see for example [11, 21, 22, 45, 55, 61]). This has led to the development of a preliminary Facilitator’s Toolkit utilising quality improvement and audit and feedback methods and also a more structured approach to the identification, training and development of facilitators within and across systems [62, 63]. (For a more detailed description of the facilitation model and toolkit, see [63]). Specifically, we are proposing a facilitation pathway from beginner or novice facilitator to experienced and expert facilitator, assuming different roles in the process of implementing and researching the implementation of new knowledge into practice [62].

Positioned as the active ingredient, facilitation is undertaken by one or more trained facilitators, who help to navigate individuals and teams through the complex change processes involved and the contextual challenges encountered. Facilitators can either be internal to the system, external to it or a combination of both, as the three case study examples illustrate, with a mix of internal-external and novice-experienced-expert combinations. This reinforces that there is not a single right way to apply facilitator roles; however, there are clear benefits in mechanisms that provide support and mentoring to new or less experienced facilitators. In case 1, this was achieved through having teams of novice and experienced facilitators working together and by bringing in novice internal facilitators to build local capacity for facilitating implementation. In case 3, facilitator pairs were formed to role model inter-disciplinary working and provide mutual support, supplemented by support from external, expert facilitators in the co-located university. In all three cases, the methods employed by facilitators typically involved improvement approaches such as Plan-Do-Study-Act cycles and audit and feedback, underpinned by project management. This helped to address key issues such as establishing clear goals, demonstrating the potential for improvement, providing regular feedback and trialing changes on a small-scale—all important factors in terms of securing and maintaining staff motivation and commitment.

The facilitator needs to have a sound understanding of the nature of the innovation being introduced (the focus and content of implementation), the individuals and teams that have to enact the change (the recipients) and the environment in which they work (the local, organisational and health system context). This essentially involves thinking about *what* is to be implemented, *who* with and *where*. Facilitation provides the *how* component of implementation.

**Fig. 1:**
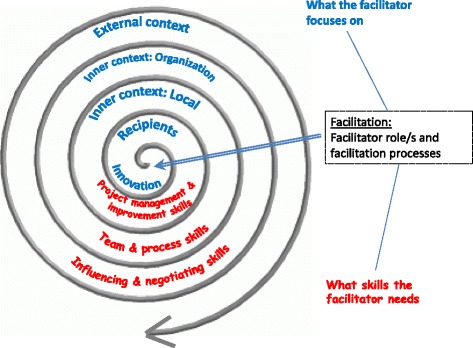
The facilitation role and process

In order to help the facilitator understand the dynamic nature of implementation, we have chosen to represent the i-PARIHS framework as a continuous spiral which starts with a focus on the innovation and the recipients, moving out to the different layers of context (inner context at local and organisational level and outer context at wider system and policy level). Figure 1 summarises what the facilitator looks at within each of these levels and also summarises the sort of activities they need to undertake; in other words, what they have to be able to do. This effectively involves progressing from a focus on the more specific, concrete aspects of implementation to addressing the contextual factors and barriers that are likely to influence the trajectory of the implementation journey. Our hypothesis is that the further out into the spiral the facilitator moves, the greater the level of experience and skill they will need. This in turn suggests that whilst novice facilitators may be able to support locally focused implementation projects (in terms of working with a local team to plan and undertake the project), they are likely to need the support of a more experienced facilitator to assess and negotiate some of the more challenging barriers or contextual factors they may encounter.

This leads us onto another important consideration about the need for facilitators to work within a supportive network, ideally mentored and supported by peers and more experienced colleagues, as is evident in the case study examples. Depending on the scale of the change being considered and how it is set up, there could be a team of facilitators, each supporting a number of units or areas. In some cases, a facilitator role may be combined with another role, such as a clinical leader, quality improvement coordinator, knowledge broker or project manager. The specific title of ‘facilitator’ is not important. The crucial thing is that the individuals function as facilitators in that they actively use facilitation methods and processes to enable and optimise implementation. In some cases—and particularly where facilitation is part of another role—the facilitator may feel like a lone agent. However, given the scope and complexity of the role, this is not a desirable situation and can result in individual feelings of isolation and being overwhelmed [62]. Whilst a formal infrastructure might not exist, the individuals concerned should be encouraged to seek opportunities for support and guidance, for example, by establishing a ‘buddy’ relationship with others in a similar role or identifying a more experienced facilitator to mentor them. Organisations committed to knowledge translation and implementing innovations in healthcare ought to reflect on the infrastructure they have to enable facilitation capabilities and skills to flourish. Otherwise, there is a danger of setting people up to fail without the requisite level of preparation, skills and support. Table 4 summarises the main descriptors of novice, experienced and expert facilitators.

**Table 4:** Novice, experienced and expert facilitators (adapted from [62])

Experience	Focus of facilitation
Novice facilitator	Working under the supervision of an experienced facilitator *Focus on:* What an innovation is; what evidence informs the innovation and how to assess and apply it Readiness to change at a local level What motivates individuals and teams and how teams work effectively What context is; what impact context has on implementation at a local and organisational level Identifying and engaging key stakeholders Planning, implementing, measuring and embedding change
Experienced facilitator	Working under the supervision of an expert facilitator *Focus on:* In depth understanding and knowledge of the organisation or organisations they are working with Awareness of competing tensions and how to manage these in relation to implementing innovation and change In depth understanding of individual and team motivation, team dynamics and productivity Experienced and knowledgeable in local context evaluation Able to assess system-wide activities and influence actions Aware of wider contextual issues and confident in terms of negotiating boundaries and political tensions
Expert facilitator	Expert facilitator operating as a guide and mentor to other facilitators *Focus on:* Coordinating and supporting networks of experienced and novice facilitators Working with health systems to improve implementation success Working across academic, service and other organisational boundaries to integrate facilitation and research activity Developing and testing theories of implementation, innovation and facilitation Evaluating implementation and facilitation interventions to generate newer knowledge Refining and improving learning materials and mentoring processes Running workshops and advanced master classes on facilitation approaches

A final point in relation to the facilitation construct within i-PARIHS is that in presenting our description of facilitation and outlining the key ingredients within the facilitator’s skill repertoire, there may be a suggestion that the process is logical and sequential. The reality, however, is very different; the interrelationship between the innovation, the recipients and the multiple layers of contexts is often unpredictable, fluid and iterative. Experienced facilitators learn how to manage this uncertainty and keep individuals and teams on track.

(Note: Additional file 1 provides a more detailed illustration of the facilitator’s focus and activities at the level of the innovation, the recipients and the multiple layers of context; Additional file 2 outlines a set of reflective questions that facilitators can use to think about key issues within the different dimensions of implementation.)

### The underpinning theoretical antecedents of the i-PARIHS framework

Another criticism of PARIHS was the lack of detail around its theoretical foundations. Unlike other frameworks such as the Knowledge-to-Action (K2A) Framework [64] and the Theoretical Domains Framework [65], which identify with a particular theoretical perspective to explain implementation (planned change and behavioural change, respectively), PARIHS claimed an eclectic provenance of relevant theories and philosophical perspectives [1]. In our deliberations with i-PARIHS, we have continued with the theoretical eclecticism but have tried to present it in a more coherent way [16]. The reason for doing this is twofold: first, it helps the facilitator to understand the theoretical antecedents of the issues they are dealing with, and second, it helps research and evaluation teams to create a theoretical framework around one or more particular aspects of the implementation process they wish to explore in greater detail. Our identification of relevant theories is necessarily selective; however, we have sought to identify those theories that reflect the core constructs of innovation, recipients, context and facilitation and that are consistent with our overarching view of implementation as iterative, negotiated and relational. Thus, if a facilitator or a research team studying implementation was interested in understanding what aspects of the evidence influenced its uptake and use, the i-PARIHS framework would point them in the direction of theories around experiential learning [66], situated learning [67], evidence-based practice [68] and innovation [34, 36, 69]. This would provide insights into the means by which knowledge is acquired, interpreted and applied in a way that is consistent with the i-PARIHS framework; in turn, it would also provide a theoretical perspective that could inform or explain the innovation and its impact.

For a facilitator thinking about how to work with individuals and groups, the i-PARIHS framework again points them towards a number of different theories. These include theories of innovation, reflecting the inter-connection between an innovation and the people who have to use it. For example, Rogers highlighted the importance of understanding different groups within the intended audience for innovation and how they are likely to react, as well as making use of peer-to-peer conversations and credible, trusted teachers and leaders to bring people on board with the change [33, 34]. Issues relating to adopter characteristics are also reflected in the Theoretical Domains Framework [65, 70] where motivation is considered alongside factors such as role and identity, goals, behavioural regulation, beliefs and capabilities and consequences. Weiner’s theory of organisational readiness to change [71] proposes that readiness depends on collective behaviour change linked to two key factors, described as change commitment (wanting to change) and change efficacy (able to change). Insights into these types of theories help to inform the way that facilitators structure their interventions to achieve the behavioural change that is required for successful implementation. Equally, they provide research and evaluation teams with a set of parameters to frame studies of implementing evidence-based innovation in practice.

Theories that inform our views about the context of implementation are rich and varied, particularly focusing on issues of organisational complexity and how organisations learn and use new knowledge. Again this is consistent with the multi-dimensional perspective of implementation that the i-PARIHS framework adopts and embedded beliefs about reflective and responsive learning. Included in this mix are theories related to complexity [72, 73], absorptive capacity [74] and learning organisations [75] as well as theories related to leadership and organisational culture [76]. Other theories relate to how innovation and change can be sustained in a system. Again, there are a number of theories that attempt to explain this phenomenon. One that has been applied in healthcare is normalisation process theory [77, 78], which acknowledges the interaction of actors (recipients) within their context and proposes four constructs titled coherence, cognitive participation, collective action and reflexive monitoring as the generative mechanisms required to routinely embed innovations. A further set of theories relevant to the study of context are economic and political theories that govern the external environment, including theories of regulation, market economy, financial incentives and contracting [79].

From this brief overview of theories, a number of common themes are apparent which reinforce the complex, dynamic and non-linear nature of implementation and emphasise the importance of experiential learning at the level of individuals, teams and organisations. What is also apparent is the relationship between aspects of the innovation, the recipients and the context. Table 5 summarises the key themes that emerge from theories relating to the ‘what’, ‘who’ and ‘where’ of implementation and resultant implications for the ‘how’ issues of implementation.

**Table 5:** Theoretical Antecedents of i-PARIHS (adapted from [16])

Focus of implementation	Themes identified from theoretical analysis	Indicative references
WHAT is being implemented: characteristics of the evidence, knowledge or innovation	Broad definitions of evidence, linked to wider literature on innovation and knowledge generation and application Embedded and emergent; influence and contribution of tacit knowledge Importance of experiential and situated learning Value of co-production	Rycroft-Malone et al. [6] Kolb [66] Lave and Wenger [67] Rogers [33, 34] Van de Ven et al. [36, 69] Greenhalgh et al. [35]
WHO is being targeted: characteristics of the target groups for implementation	Recognition of ‘want to’ and ‘can do’ factors (motivation and capability/capacity) Importance of collectivity and learning within communities Different responses to innovation and change Different learning styles Existence of boundaries between different groups/communities Increasingly complex boundaries as innovation increases in novelty Influence of social networks	Rogers [33, 34] Weiner [71] Michie et al. [70] Cane et al. [65] Wenger [87] Gabbay et al. [39, 88] Carlile [89]
WHERE: characteristics of the setting in which implementation takes place	Organisations as complex, adaptive systems Emphasis on learning at the individual, team and organisational level Influence of culture and mental models Influence of prior knowledge and experience Importance of collaboration, coordination and networks for knowledge exchange	Plsek and Greenhalgh [72] Argyris and Schon [90] Senge [75] Schein [76] Grol et al. [79] Harvey et al. [74]
HOW: implications for the process of implementation	Distributed learning – through teams and networks Importance of flexibility and adaptability Tailoring approaches to different needs and responses Reflective learning Credible and trusted leaders and teachers Distributed/shared leadership Building relationships Understanding and communicating practices	Rogers [82] May and Finch [77] Heron [83] Deming [84] McKee et al. [91]

The final groups of theories informing the i-PARIHS framework are those that inform our views about facilitation. As Table 5 illustrates, the themes identified from our theoretical analysis have important implications for ‘how’ the process of implementation is approached. Our position is that the concept of facilitation, with its emphasis on enabling others to act, is an ideal way in which to embrace processes that recognise and adapt to the dynamic and situation-specific nature of implementation, with an emphasis on building relationships, learning and flexibility. As others have noted, there is still more work to do on clarifying the concept of facilitation [80, 81]. The theories that have particularly influenced our approach to facilitation include those of humanist authors such as Carl Rogers [82] and John Heron [83]. Fundamentally, this theoretical perspective on facilitation emphasises the importance of enabling others, as opposed to telling, teaching, persuading or coercing them to act. We also draw on improvement theories that promote local engagement and ownership of the process of implementing improvement, particularly in thinking about how facilitators enact their role in practice. Most notable amongst these improvement theories is Deming’s system of profound knowledge for improvement, with its focus on understanding systems, processes, experiential learning and human interaction [84].

### Where next?

Our analysis of a range of theoretical, empirical and experiential evidence gives us initial confidence in the revisions proposed in the i-PARIHS framework. However, just as PARIHS has evolved over time, so too we recognise the need for ongoing development and evaluation of i-PARIHS. Our aim in presenting the framework at this point is to open up further discussion and debate. Some specific issues that we would suggest merit further consideration include the re-conceptualisation of the core constructs. From the discussion in the paper, we have tried to delineate the boundaries between the constructs of innovation, recipients and context; yet we know that, depending on the situation, there may be overlap between them thus any attempt to distinguish them may be imperfect and open for debate. From a pragmatic perspective, we have attempted to reflect the inter-connection of the constructs in the spiral representation of i-PARIHS yet at the same time provide practical guidance to those involved in implementation. Whether this is a helpful distinction will depend on feedback from future users of the framework. Likewise, we recognise that the labelling of the construct ‘recipients’ may present a rather passive role for the actors involved in implementation. A more active descriptor may be appropriate, particularly to reflect the role of stakeholders—and most importantly patients and clients—in shaping the innovation and implementation process.

We have addressed some of the challenges set out in the 2008 paper in Implementation Science, notably the need for more theoretical work on PARIHS and more detail on how to operationalise the framework [1]. Clearly, future work is required to test and refine the proposed i-PARIHS framework as both a diagnostic and evaluative tool within implementation practice and implementation research, particularly through prospective implementation studies. Within such studies, it will be important to adopt research and evaluation designs that allow in-depth investigation of the core constructs. The inter-play between constructs that influence implementation is generally poorly understood, not least due to the problems of boundary delineation mentioned above; as such, there is a need for more in-depth longitudinal studies which examine the dynamics of the innovation, the actors involved, the context and the proposed model of facilitation. This in turn, will inform the ongoing development and refinement of instruments to be used in conjunction with i-PARIHS and its core constructs. In parallel, ongoing work to map, apply and evaluate the theoretical antecedents of the framework is warranted, particularly to further clarify and then evaluate the effectiveness of facilitators and facilitation as an intervention for knowledge translation.

## Conclusions

The PARIHS conceptual framework was developed in an attempt to represent the dynamic and multi-faceted nature of implementation in healthcare. The framework has been widely applied, tested, reviewed and refined. Drawing on evidence from our own and others’ experiences of applying and evaluating PARIHS, this paper presents a revised version of the framework, described as the integrated-PARIHS or i-PARIHS framework. This reflects the work that has been undertaken to explicitly integrate the conceptual framework with supporting theories and an operational model of facilitation. The revised i-PARIHS framework positions facilitation as the active ingredient of implementation, assessing and aligning the innovation to be implemented with the intended recipients in their local, organisational and wider system context. Facilitation is operationalised through a network of novice, experienced and expert facilitators applying a range of enabling skills and improvement strategies to structure the implementation process, engage and manage relationships between key stakeholders and identify and negotiate barriers to implementation within the contextual setting. We are presenting the revised framework for consideration and debate within the wider implementation science community, recognising that future work is needed to test its utility, applicability and content and construct validity.

## Additional files

Additional file 1: Detailed illustration of the facilitator’s focus and activity at the level of the innovation, the recipients and the inner and outer context. This figure provides further information on specific issues the facilitator may need to consider when planning for implementation. (PPTX 276 kb)

Additional file 2: Reflective questions that facilitators can use to think about key issues within the different dimensions of implementation. This file presents a tool for self-assessment that facilitators can use to consider their own strengths and areas for development in supporting implementation. (PDF 205 kb)
