# Supplementary material for: PARIHS revisited: from heuristic to integrated framework for the successful implementation of knowledge into practice
Source: Implement Sci. 2016 Mar 10;11:33. doi: 10.1186/s13012-016-0398-2 (PMC4807546; doi:10.1186/s13012-016-0398-2)
Supplement: Supplementary file 1 — Detailed illustration of the facilitator’s focus and activity at the level of the innovation, the recipients and the inner and outer context. This figure provides further information on specific issues the facilitator may need to consider when planning for implementation. (PPTX 276 kb) [file 13012_2016_398_MOESM1_ESM.pptx]

## Slide 1
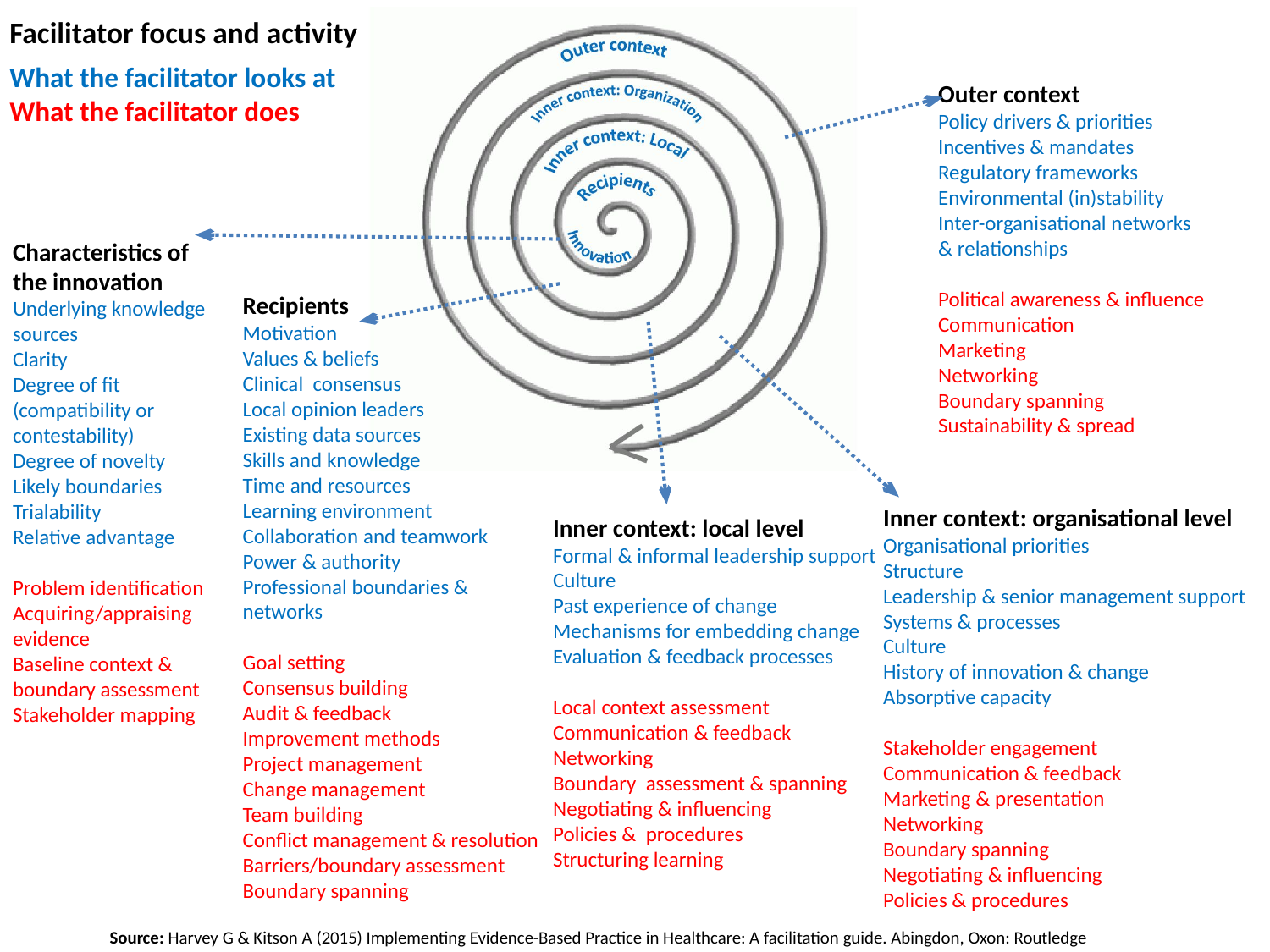

Facilitator focus and activity
What the facilitator looks at
What the facilitator does
Outer context
Policy drivers & priorities
Incentives & mandates
Regulatory frameworks
Environmental (in)stability
Inter-organisational networks & relationships
Political awareness & influence
Communication
Marketing
Networking
Boundary spanning
Sustainability & spread
Characteristics of the innovation
Underlying knowledge sources
Clarity
Degree of fit (compatibility or contestability)
Degree of novelty
Likely boundaries
Trialability
Relative advantage
Problem identification
Acquiring/appraising evidence
Baseline context & boundary assessment
Stakeholder mapping
Recipients
Motivation
Values & beliefs
Clinical consensus
Local opinion leaders
Existing data sources
Skills and knowledge
Time and resources
Learning environment
Collaboration and teamwork
Power & authority
Professional boundaries & networks
Goal setting
Consensus building
Audit & feedback
Improvement methods
Project management
Change management
Team building
Conflict management & resolution
Barriers/boundary assessment
Boundary spanning
Inner context: organisational level
Organisational priorities
Structure
Leadership & senior management support
Systems & processes
Culture
History of innovation & change
Absorptive capacity
Stakeholder engagement
Communication & feedback
Marketing & presentation
Networking
Boundary spanning
Negotiating & influencing
Policies & procedures
Inner context: local level
Formal & informal leadership support
Culture
Past experience of change
Mechanisms for embedding change
Evaluation & feedback processes
Local context assessment
Communication & feedback
Networking
Boundary assessment & spanning
Negotiating & influencing
Policies & procedures
Structuring learning
Source: Harvey G & Kitson A (2015) Implementing Evidence-Based Practice in Healthcare: A facilitation guide. Abingdon, Oxon: Routledge
