# Supplementary material for: PARIHS revisited: from heuristic to integrated framework for the successful implementation of knowledge into practice
Source: Implement Sci. 2016 Mar 10;11:33. doi: 10.1186/s13012-016-0398-2 (PMC4807546; doi:10.1186/s13012-016-0398-2)
Supplement: Supplementary file 2 — Reflective questions that facilitators can use to think about key issues within the different dimensions of implementation. This file presents a tool for self-assessment that facilitators can use to consider their own strengths and areas for development in supporting implementation. (PDF 205 kb) [file 13012_2016_398_MOESM2_ESM.pdf]

Planning for Implementation: A facilitation checklist

When preparing for an implementation project, there are a number of factors that are important to assess – factors that relate to the innovation or change that you are trying to introduce; factors linked to the individuals and teams that you want to adopt the change; and factors concerned with the wider environment, both internally and externally to the organization in which implementation is taking place. Use the set of reflective questions below to help you assess and diagnose where you might need to focus attention in terms of facilitating the implementation process.

1. Characteristics of the innovation

- Who is likely to be affected by the proposed innovation?
- What is the underlying evidence for the proposed innovation or change?
    - Is it derived from research, clinical consensus, patient views, local information/data – or a combination of these?
    - Is it viewed as rigorous and robust?
    - Is there a shared view about the evidence?
    - How well does it 'fit' the local setting?
    - Is it likely to be accepted or contested by those people who have to implement it?
- Is the evidence packaged in an accessible and usable form e.g. a clinical guideline, care pathway or algorithm?
    - Will people be able to see easily and clearly what is proposed in terms of clinical practice and the process of patient care?
- How much novelty does the evidence introduce?
    - Will it require significant changes in the processes and/or systems of care delivery?
    - Will it present a challenge to people's ways of thinking, mental models and relationships?
    - What are the implications of this in terms of the likely boundaries that will be encountered?
    - Will a knowledge transfer, translation or transformation strategy be required?
- Does it offer advantages over the current way of doing things, for example:
    - Will it enhance patient experience?
    - Could it introduce greater efficiency in the provision of care?
    - Will it help to remove bottlenecks in the care process?
- Is there potential to test out/pilot the introduction of the evidence/innovation on a small scale in the first instance?

2. The recipients of the evidence/innovation

Think about the people who you want to implement the change and how they are likely to respond - both at an individual level and as a member of a clinical or service delivery team. Reflect on whether they want to introduce the innovation and if they are able to implement the required changes.

<u>2a. Motivation to change: Individual level</u>

- Do individual members of the team want to apply the change in practice?
- Do they perceive the proposed change as valuable and worthwhile?
- Do they see a need to make the change?
- Is the change consistent with their existing values and beliefs?
- Are there individuals who function as local opinion leaders? Will they be supportive or obstructive in terms of introducing the proposed change?

<u>2b. Motivation to change: Team level</u>

- At a collective level, does the team want to apply the change in practice?
- Is the proposed change seen as valuable and worthwhile?
- Do they see a need to make a change?

- Is there a shared view or are there differences of opinion e.g. between key individuals or between different professional groups and communities of practice?
- Is there existing data that can be used to highlight the potential for improvement? Or can you collect data for this purpose?

<u>2c. Ability to change: Individual level</u>

- Are individual members able to implement the proposed change?
  - Do they understand what the change entails?
  - Is it within their current level of knowledge and skills?
  - Will additional training and development be needed?
- Do people understand the modifications that will needed to routine practice and how to change and embed these?
- Do individuals have the necessary authority to carry out the proposed changes?
- Have key individuals whose support is needed been identified? Are they engaged in discussing and planning implementation?

<u>2d. Ability to change: Team level</u>

- Are the team able to implement the proposed change?
  - Do they understand what the change entails?
  - Is it within their current level of knowledge and skills?
  - Will additional training and development be needed?
- Does the team understand the modifications that will needed to routine practice and how to change and embed these?
- Does the team have the necessary authority to carry out the proposed changes?
- Is there good inter-professional collaboration and team-work – between professional groups and between clinical staff and managers?
- Will support be needed to develop more effective collaboration and team-work?
- Are the potential barriers to implementation known? Are there strategies in place to address these?
- Are the resources available to support the implementation process, for example: time and/or financial support for new skills development, new equipment, expert support and advice?

3. The inner context

Think about the characteristics of the context in which the innovation is to be implemented – both the immediate local context in which the recipients are working and the wider organizational context in which their unit or department operates.

<u>3a. The local context</u>

- Who are the formal and informal leaders at a local level?
  - Are they likely to be supportive of the proposed change?
  - Are the leaders helping to create a facilitative context through providing motivation and support, creating a vision and reinforcing the change process?
  - Is there a distributed and devolved style of management?
- Is there a culture that supports innovation and change?
  - Do staff feel actively involved in decisions that affect them?
  - Are staff trusted to introduce new ideas into practice?
  - Do staff and patients feel valued?
- What is the past experience of introducing changes at a local level?
- Are there mechanisms in place to support learning and evaluation and to embed changes in routine practice e.g. regular team meetings, audit and feedback processes, professional development opportunities and performance review systems?

3b. The organizational context

- Do the evidence/innovation and the changes proposed align with the strategic priorities for the organization?
- Has the support of key individuals and leaders within the organization been sought and secured?
- Is there a culture that supports innovation and change?
  - Is there a history of successful and sustained change within the organization?
- Does the organization have systems and processes in place that support innovation and change e.g. effective information and communication systems, opportunities for networking and learning across departments/teams?
- Do the senior management team actively seek opportunities for improvement and encourage ideas and feedback from patients, the public and staff?
- Are there mechanisms in place for embedding changes in routine practice e.g. formal policies and procedures?

4. The outer context

Consider what is happening in the wider health system that might affect the inner context. Whilst it may not be possible to directly influence the outer context, it is important to be aware of how the outer context might impact upon local implementation - and whether this creates opportunities from which the project might be able to gain leverage.

- Do the evidence/innovation and the changes proposed align with the strategic priorities for the wider health system e.g. in terms of current health policy, national priorities for action and improvement?
- Are there incentives in the wider health system that reinforce the proposed change e.g. pay for performance schemes, regulatory requirements etc.?
- Are there inter-organizational networks (e.g. specialised clinical networks) that will be helpful in terms of supporting the proposed changes?
- How much stability/instability is there in the wider health system?
  - Is this likely to influence the implementation project?

Source: Harvey, G and Kitson, A. Implementing Evidence-Based Practice in Healthcare: A facilitation guide. Abingdon, Oxon: Routledge, 2015.
